# The role of morphology and coupling of gold nanoparticles in optical breakdown during picosecond pulse exposures

**DOI:** 10.3762/bjnano.7.79

**Published:** 2016-06-16

**Authors:** Yevgeniy R Davletshin, J Carl Kumaradas

**Affiliations:** 1 Department of Physics, Ryerson University, Toronto, ON, M5B 2K3, Canada

**Keywords:** electron plasma, finite element method, optical breakdown, plasmon coupling, plasmonic nanoparticles

## Abstract

This paper presents a theoretical study of the interaction of a 6 ps laser pulse with uncoupled and plasmon-coupled gold nanoparticles. We show how the one-dimensional assembly of particles affects the optical breakdown threshold of its surroundings. For this purpose we used a fully coupled electromagnetic, thermodynamic and plasma dynamics model for a laser pulse interaction with gold nanospheres, nanorods and assemblies, which was solved using the finite element method. The thresholds of optical breakdown for off- and on-resonance irradiated gold nanosphere monomers were compared against nanosphere dimers, trimers, and gold nanorods with the same overall size and aspect ratio. The optical breakdown thresholds had a stronger dependence on the optical near-field enhancement than on the mass or absorption cross-section of the nanostructure. These findings can be used to advance the nanoparticle-based nanoscale manipulation of matter.

## Introduction

Over the last decade the interaction of pulsed lasers with gold nanoparticles has been studied in many emerging fields, such as sensing and medical diagnostics and therapy [1–2]. This interest is sparked by the ability to overcome diffraction-limited optics and to control electromagnetic field interactions at the nanoscale using nanoparticles. This relies on the unique tunable optical properties of gold nanoparticle stemming from the interaction of light with the quasi-free electrons in gold. The oscillation of these electrons induces surface plasmon resonance, resulting in the enhancement of the incident electric field in the vicinity of the nanoparticle. This can result in secondary phenomena associated with laser pulse interaction, such as heating of the surrounding media, acoustic wave formation and optical breakdown. The surface plasmon resonance is tunable over a wide range of frequencies and affected by the optical properties of the surrounding environment [3], the morphology of individual nanoparticle as well as the formation of nanoparticle assemblies [4–5].

Nanospheres and nanorods are the two most common shapes of gold nanoparticles. They can be made to assemble through binding to the surface of cell [6–9], modification of gold nanoparticle surfaces to cause binding to each other [10–11] or endocytosis [12]. Nanoparticle assemblies exhibit optical behavior that differs from single particles. The changes in the optical behavior of nanoparticle assemblies are governed by surface plasmon resonance coupling that occurs at distances of less than 2.5 diameters between the surfaces of two adjacent nanoparticles [4]. This effect depends on the orientation, spacing and shape of the adjacent nanoparticles [9,13–15]. The plasmon coupling effect broadens and shifts the plasmon resonance peaks of gold nanospheres towards the near infrared region. (This is useful in biological applications, where light has a good penetration depth) [5,16].

The use of plasmonic nanoparticles and the associated near-field enhancement has been used in applications based on the laser-induced breakdown (LIB) effect [17]. Nanostructure fabrication [18], cell nanosurgery [19], and laser-induced breakdown spectroscopy [20–21] are applications where the use of nanoparticles for LIB enhancement has produced promising results. In biomedical applications, nanoparticle-mediated LIB is a non-linear laser energy absorption process that produces a low-density plasma in the surrounding region of the nanoparticle. Plasmonic nanoparticles such as gold nanospheres and nanorods, enhance the electric field of the incident light and strongly absorb the light. LIB is usually induced when the laser irradiance surpasses a certain threshold (which will henceforth be referred to as the “optical breakdown threshold”) and the free-electron density exceeds a critical value in the range of 10^18^–10^21^ cm^−3^ [17,22–26]. The interaction of a strong electromagnetic field with a gold nanoparticle in an aqueous medium, which models a biological environment, can initiate breakdown (i) through multiphoton absorption and the tunneling effect, usually referred to as laser-induced optical breakdown (LIOB) [26–27]; (ii) through a thermal initiation pathway also known as laser-induced thermal breakdown (LITB) [28–29]; or (iii) through the photo-thermal emission of hot electrons off the surface of the nanoparticle [30–31]. After some seed electrons have been generated via a combination of the processes mentioned above, the plasma starts to gain sufficient kinetic energy from the laser pulse by inverse Bremsstrahlung absorption and grows through impact ionization known as electron avalanche [32]. LIB induces breakage of molecular and atomic bonds and will be accompanied by luminescence, cavitation, and the formation of bubbles and shock waves [22,33].

The optical breakdown threshold in an aqueous medium that contains gold nanoparticles is a function of the ionization energy, the impurity level of the medium, nanoparticle morphology and concentration, and the laser beam characteristics (pulse duration, wavelength and intensity) [19]. In an aqueous medium without nanoparticles, the initiation of multiphoton absorption for LIOB will require between six and twelve photons with the same polarization to exceed the band-gap energy of water, which is approx. 6.5 eV [34]. LIOB occurs when the optical breakdown threshold, which is in the range of about 10^11^–10^13^ W/cm^2^, is surpassed in the focal region of the laser beam [34–35]. For nanosecond pulses [28] or ultrashort pulse sequences separated by times of the order of the free-electron energy thermalization time [36], LITB is a significant source of seed electrons, ρ_seed_, which is required to initiate the avalanche ionization [28], when the temperature of the medium increases above approx. 5000 K. LITB starts with linear absorption and thermal ionization of the aqueous medium and continues with non-linear avalanche ionization. The introduction of gold nanoparticles into an aqueous medium will lower the LIB threshold and provide an additional source of seed electrons from a photo-thermal emission of hot electrons off the surface of the nanoparticles [31,37]. During nanoparticle-mediated LIOB with pulses shorter than 10 ps the lattice temperature of the nanoparticle is kept below the melting point (1337 K for gold nanoparticles with a diameter above 10 nm [38]) since the photon energy at this time-scale is transferred to the electrons in the gold nanoparticle and diffusion is minimized [39–40]. The gold nanoparticles therefore retain their optical properties during LIB with pulses shorter than 10 ps. Compared to femtosecond lasers, ultrashort picosecond lasers (with pulse durations between 1 and 10 ps) are beneficial due to their cheaper operational cost, ease of maintenance and higher power specifications. Such ultrashort pulses (few picoseconds) can be achieved via using Nd:YAG and Nd:YVO4 crystals in passively modulated mode-locked lasers, which are widely used in micromachining [41]. For our calculations we have used a pulse duration of 6 ps due to availability of such picosecond lasers from a variety of suppliers, such as: FORC-Photonics, LEUKOS, TOPTICA Photonics AG, Onefive GmbH, Time-Bandwidth Products AG, Alnair Laboratories Corp, Clark-MXR Inc., Calmar Laser, Atseva LLC, Fianium LTD, EKSPLA.

The use of nanoparticle-mediated LIB is complicated due to its non-linear nature. The variety of parameters, such as morphology and nanoparticle assembly (plasmon coupling) that influence its behavior motivate a theoretical description of the optical breakdown process [37,42–43]. Hatef and Meunier [43] recently reported the impact of size and inter-particle distance for femtosecond laser pulse widths on the energy absorption by gold nanosphere dimers and by the plasma surrounding the dimers. They showed that the energy deposition in the plasma increases with decreasing gap distance between dimers down to a 4 nm gap, which was the smallest gap they examined. Boulais et al. [37] revealed the existence of two different physical regimes of plasma generation in the vicinity of a gold nanorod during ultrafast pulse exposure. For a fluence lower than 3 mJ/cm^2^, the gold nanorod strongly absorbed the incident pulse energy and the majority of the seed electrons were produced by photo-thermal emission (the absorption regime), while for fluences higher than 3 mJ/cm^2^ the formation of free electrons was dominated by multiphoton absorption due to a high near-field enhancement surrounding the gold nanorod (the near-field regime). At picosecond pulses with low irradiation fluence, nanoparticle-mediated LIB is dominated by photo-thermal emission due to the fast temperature increase of the electrons in the nanostructure.

The lack of a detailed understanding of the mechanism of plasma formation in the vicinity of gold nanoparticles in the picosecond regime hinders the interpretation of experimental results and the development of cell transfection mediated through gold nanoparticles [44–45]. For applications such as transfection a complete theoretical picture of plasma formation in the picosecond regime with the use of gold nanoparticles is needed to optimize its use [44]. This will lower the operating cost compared to transfection using femtosecond pulsed lasers. The lack of understanding of how the morphology and assembly of gold nanoparticles lowers the optical breakdown threshold also hinders the design and optimization of gold nanoparticles in LIB to target specific cells based under the chemical and physical conditions of the environment [10,46].

In this paper, we present a theoretical investigation of the role of the morphology of gold nanoparticles on the optical breakdown threshold. We present a comparison of on- and off-resonance 6 ps laser pulse interactions with uncoupled and tightly-coupled gold nanospheres and nanorod monomers of different sizes, with a focus on the thermal and optical processes. The role of nanoparticle morphology and plasmon coupling in low-density plasma generation is analyzed. This was done by comparing nanosphere monomers against nanosphere dimers and trimers and against nanorods having the same aspect ratio and size, as the nanosphere dimers and trimers. We conclude that the nanoparticle-mediated LIB threshold in the picosecond regime is highly dependent on the optical near-field enhancement instead of nanoparticle size and absorption cross-section. The findings of this study will help in LIB-related fields to advance the understanding of nanoparticle–laser interactions, which will lead to the better design of experiments by accounting for all related optical and thermal effects.

## Methods

To model the generation of a low-density plasma through a picosecond laser pulse in the vicinity of gold nanoparticles one should account for several physical phenomena that happen during the pulse, such as: the interactions of electromagnetic fields with gold nanoparticles and their environment, the absorption of the pulse energy by the nanoparticles and their environment and the resulting heating, and the generation of a free-electron plasma and its effect on the optical properties of the environment. The generation of a free-electron density above 10^18^ cm^−3^ during the pulse will change the optical properties of the environment in the vicinity of the nanoparticle, shielding the nanoparticle from the incoming irradiation. This will lead to a lowering of the absorption cross-section of the nanoparticle, lowering its heat generation. Photo-thermal emission of hot electrons off the nanoparticle surface provides a significant number of seed electrons that can trigger avalanche ionization.

Two theoretical models have been used to model the plasma generation in the vicinity of gold nanoparticles. The model by Bisker and Yelin [42] provided a theoretical analysis of short pulse interactions with spherical noble-metal nanoparticles, while Boulais et al. [31] developed a model of plasma-mediated nanocavitation for gold nanospheres and nanorods [31,37]. The model by Bisker and Yelin [42] is based on Mie theory and rate equations for free-electron density generation [26,34]. This model does not have a full two-way coupling between the Mie simulations and free-electron plasma rate equations and omits the photo-thermal emission of hot electrons off the nanoparticle surface [30]. In addition the Mie theory is not applicable to arbitrarily shaped objects and cannot be used with plasmon-coupled systems. The model by Boulais et al. [31] is more complete and is based on the finite element method where all physical phenomena can be fully two-way coupled. The model includes the electromagnetic interaction of the laser pulse with gold nanoparticles, thermodynamic calculations based on the Boltzmann semiclassical transport equation [47], plasma generation described by Keldysh theory of multiphoton ionization [34], impact ionization [48], and thermo-assisted photo-thermal emission from the particle [30]. Although the model by Boulais et al. [31] is complete and can be used for arbitrarily shaped objects and plasmon-coupled nanoparticles it does not include the thermal ionization of the aqueous medium. Thermal ionization plays an important role for short pulses (picoseconds and nanoseconds) and ultrashort (femtoseconds) pulse sequences [28,36]. The model also does not include the size corrected dielectric function of gold [3].

In order to overcome the limitations of these models, we combined a fully coupled theoretical model of the electromagnetic field interaction with a gold nanoparticle, which includes (i) the surface confinement corrections to the bulk optical properties of gold [3], (ii) a hyperbolic two-temperature model for the thermodynamic evolution of the electron and lattice temperatures of the gold nanoparticle [47], (iii) a rate equation of free-electron plasma generation in an aqueous environment based on the Keldysh theory of multiphoton ionization, the tunnel effect, the avalanche and thermal ionization of water [28]; and (iv) the photo-thermal emission of hot electrons off a surface of a gold nanoparticle [30]. A summary of the model is provided below, while the complete theory with parameters used is provided in Supporting Information File 1.

### Electromagnetic (EM) model

**Figure 1:**
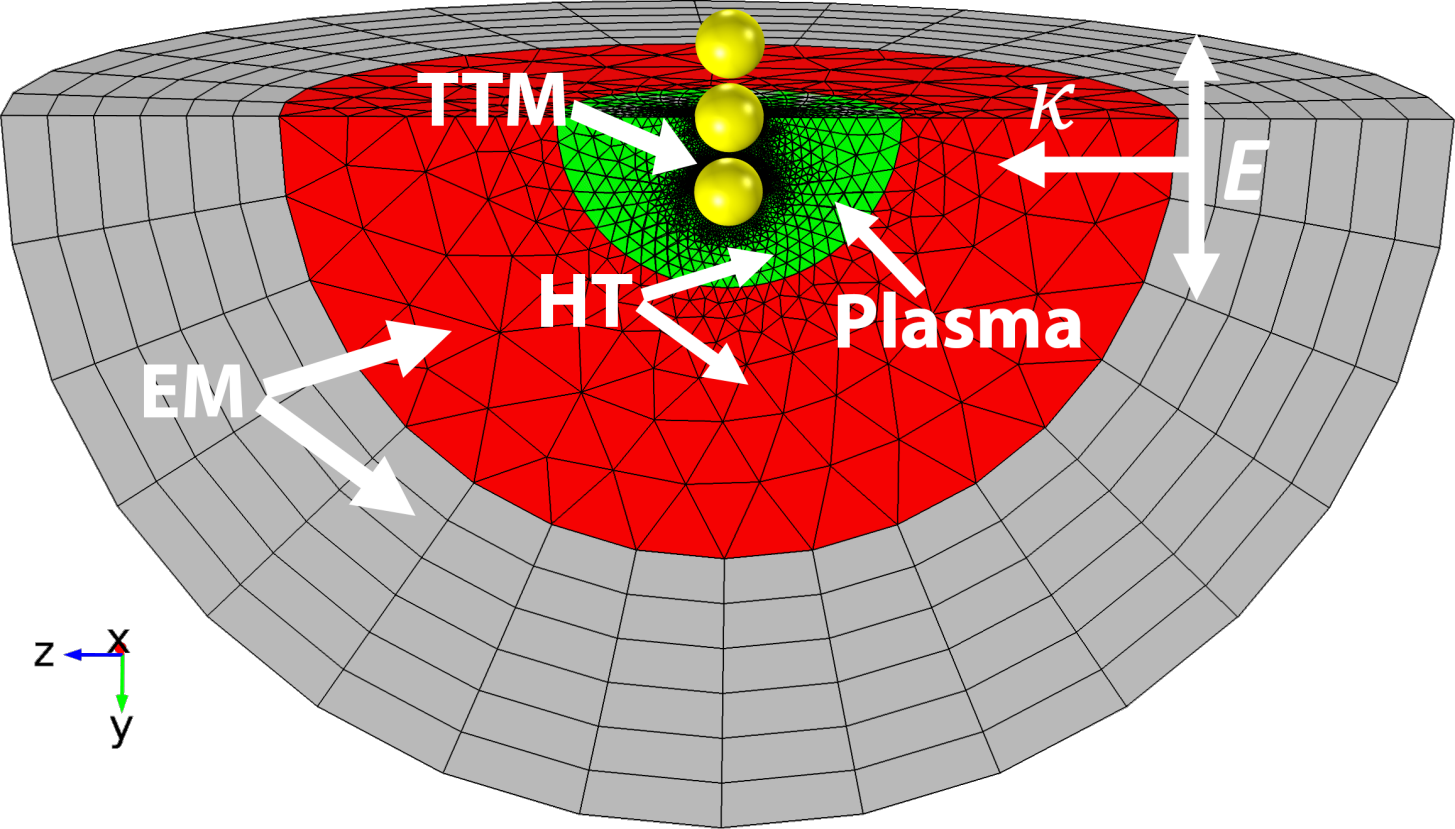
Schematic of the model geometry for a nanosphere trimer. The model contains three concentric domains. The most outer domain (grey) represents a combination of a perfectly matched layer (PML) domain and an absorbing boundary condition to truncate the electromagnetic domain and reduce reflections from artificial boundaries. The red and green domains represent an aqueous environment with dielectric and physical properties of water. The green domain is used to calculate the plasma formation, and the green and red domains are used to calculate the heat transfer in the medium. The yellow domain shows the gold nanostructure geometry. The incident electric field is linearly polarized along the nanosphere trimer length (*y*-axis) and propagates parallel to the positive *z*-axis.

The electromagnetic field was calculated using the homogeneous Helmholtz wave equation in all domains (see Figure 1). The incident electric field was linearly polarized along the longest axis of the nanostructure (*y*-axis in Figure 1) with the propagation parallel to the positive *z*-axis. Perfect magnetic conductor (PMC) and perfect electric conductor (PEC) boundaries as well as an absorbing boundary condition using perfectly matched layers ”PMLs” were used to reduce and truncate the geometry [49]. The bulk properties of the medium in the EM domain, were set to a refractive index of 1.4 [50] to better mimic the optical properties of tissue environment, where it varies from 1.34 to 1.55 [50–52]. The refractive index of the immediate vicinity of the nanoparticles can also change significantly. This can be affected by binding polymers or intracellular molecules. We tested the effect of changes in the refractive index of the vicinity of a nanostructure by adding a 2 nm shell with a refractive index of 1.6.

The dielectric function of water surrounding the nanostructure were modeled using the Drude formalism (see Supporting Information File 1) in order to account for plasma formation and the shielding of the nanostructure from the incident irradiation. The optical properties of gold with size corrections to the bulk dielectric function were used for the nanostructures [3]. Since the size corrections to the bulk dielectric function of gold may significantly alter the near-field enhancement and absorption cross-section of small nanoparticles (with diameters below 20 nm), the effect of including the size-corrected dielectric function was assessed using the model.

### Two-temperature model (TTM)

A hyperbolic two-temperature model for the evolution of the electronic and lattice temperatures of gold nanoparticles and the finite heat diffusion at the gold–water interface during the laser pulse was solved using the parameters given by Chen and Beraun [47]. The TTM was coupled to the EM model through the resistive losses during laser pulse interaction with the gold nanostructures, *Q*_rh_ [49].

### Heat transfer (HT)

The temperature produced by heat sources due to plasma formation [28], laser pulse interaction and thermal diffusion was solved in all domains outside of the nanoparticles, except the PMLs (Figure 1). The HT model was coupled to the TTM using the heat diffusion from the gold lattice to the surrounding medium through interface conductance, *Q*_au|w_ [53].

### Plasma dynamics

The dynamics of plasma formation was calculated in a spherical domain surrounding the nanoparticles (Figure 1). The plasma rate equation [28], based on the full Keldysh theory for multiphoton ionization [54], the tunneling effect, avalanche ionization, thermal ionization [28,36], diffusion, and recombination losses and photo-thermal emission (PTE) [30] of hot electrons from the gold surface, was solved to determine the dynamics of the free-electron plasma density in the vicinity of the nanoparticle. The parameters for plasma theory are those described by Linz et al. [28] and Bulgakova et al. [30]. The plasma dynamics model was coupled to the EM model through the electric field, and the changes in the dielectric function of the environment due to the free electron plasma formation. The temperature rise of the conduction electrons in the gold from the TTM were used to couple TTM to the photo-thermal emission of hot electrons. The HT model was coupled to PTE of the plasma dynamics model through the temperature rise in the medium.

The thermal ionization in water starts to play a significant role in the production of free electrons when the temperature in the focal volume of the laser is above 5000 K [28,36], and large free-electron densities are already achieved by multiphoton and cascade ionization. On the other hand, thermal ionization also plays a role during repeated sequences of ultrashort laser pulses, when the time delay between pulses is of the order of the thermalization time of the electron plasma energy [36]. Thermal ionization also partially depletes the density of bound electrons in the valence band and reduces the rate of multiphoton and impact ionization [28]. In our model, the temperature of the medium during the single-pulse irradiation is lower than 5000 K, yet we still incorporated a thermal ionization term, based on derivations given by Linz et al. [28], into the free-electron density rate equation (see Supporting Information File 1) for future studies of ultrashort pulses with a high repetition rate, where thermal ionization is significant.

Photo-thermal emission of hot electrons on the other hand, must be taken into account of free-plasma generation during 6 ps pulse exposure, since we are simulating the so-called low-fluence absorption regime [37] and the temperature of the electrons of the nanoparticle rises to a level where the electrons can cross the energetic barrier between metal and medium of *W*_au_ = 3.72 eV and contribute to the formation of an electron-density plasma [55].

These models were implemented and coupled together using the commercially available finite element package, COMSOL Multiphysics version 4.4. COMSOL Multiphysics is able to solve coupled partial differential equations and ordinary differential equations for arbitrary geometries in the time and frequency domains. The 3D model was built to solve for the electromagnetic (EM) wave propagation, the hyperbolic two-temperature model (TTM) of finite-speed heat diffusion inside gold nanoparticles, the heat transfer in aqueous media and the rate equation of plasma formation. The particles in an assembly were spaced 4 nm apart in order to account for separation by surfactant or coupling molecules on the particles surface while still keeping a strong plasmonic coupling effect [43]. The 3D geometry was reduced to one quarter of the full geometry (see Figure 1) by utilizing symmetry planes and absorbing boundary conditions. PEC and PMC boundaries are used to truncate the domain to one quarter of the full 3D EM model. The TTM, plasma and heat transfer domains were meshed using tetrahedral elements with quadrilateral vector basis function (see Figure 1). Swept meshing was used for the EM domain (see Figure 1), which represents a perfectly matched layer (PML). The maximum mesh element size was kept below one tenth of the incident wavelength in water with at least ten times finer elements in the plasma and TTM domains (Figure 1). An iterative geometric multigrid solver was used to solve for the electric field. The direct PARDISO solver with a nested dissection multithreaded algorithm was used for all other models. The coupled model was solved using the frequency-transient stepping with a second order backward differentiation after applying a global scaling, with a tolerance of 0.001.

## Results and Discussion

The interaction of a 6 ps laser pulse with gold nanoparticles in an aqueous environment was simulated for the nanostructures listed in Table 1.

**Table 1:** Gold nanoparticle types and dimensions used in the model. The letters “s”, “r”, “m”, “d” and “t” stands for sphere, rod, monomer, dimer and trimer, respectively. The number defines the radius of the nanosphere or nanorod in nanometers. The gold nanorods were modeled having an overall size of the corresponding nanosphere assembly with matching aspect ratios. For nanosphere assemblies the inter-particle distance, edge to edge, was kept at a constant value of 4 nm. The nanoparticle volume, *V*_np_, for gold nanosphere assemblies is given as the sum of the individual nanoparticle volumes.

nanostructure	aspect ratio	overall size (nm)	*V*_np_ (× 10^3^nm^3^)

nanospheres			

s5m	1:1	10 × 10	0.52
s15m	1:1	30 × 30	14.14
s25m	1:1	50 × 50	65.45
s5d	2.4:1	24 × 10	1.05
s15d	2.13:1	64 × 30	28.27
s25d	2.08:1	104 × 50	130.90
s5t	3.8:1	38 × 10	1.57
s15t	3.26:1	98 × 30	42.41
s25t	3.16:1	158 × 50	196.35

nanorods			

r5d	2.4:1	24 × 10	1.62
r15d	2.13:1	64 × 30	38.17
r25d	2.08:1	104 × 50	171.48
r5t	3.8:1	38 × 10	2.72
r15t	3.26:1	98 × 30	62.20
r25t	3.16:1	158 × 50	277.51

**Figure 2:**
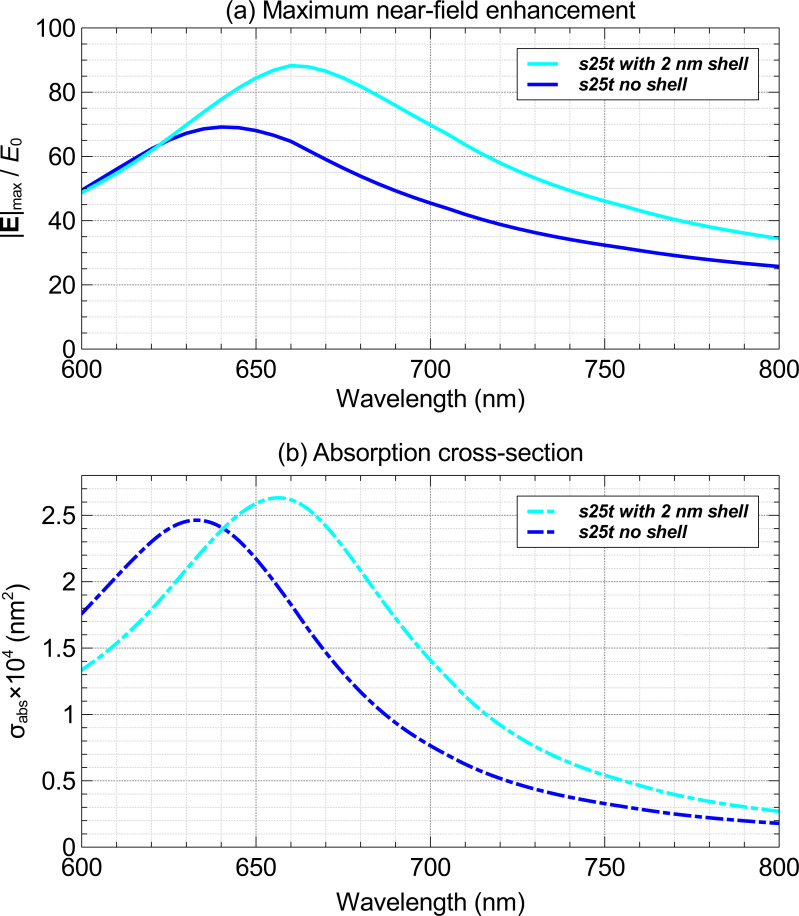
Maximum near-field enhancement, |**E**|_max_/*E*_0_, and absorption cross-section, σ_abs_, of a 25 nm gold nanosphere trimer with and without inclusion of 2 nm thick shell with a refractive index of 1.6.

Figure 2 shows the change in the optical response of the 25 nm gold nanosphere trimer (s25t) when adding a hypothetical 2 nm thick homogeneous layer with a refractive index of 1.6 around it. There is a 20 nm shift in the resonance wavelength for maximum near-field enhancement, |**E**|_max_/*E*_0_, and absorption cross-section, σ_abs_, and there is a 28% increase in |**E**|_max_/*E*_0_, and a 9% increase in σ_abs_ at the resonant wavelength due to the addition of the 2 nm thick layer around the nanospheres. Such changes in the optical response by the nanostructure will affect multiphoton absorption by the environment in the vicinity of gold nanoparticle and photo-thermal emission by the gold nanoparticle. Ideally, these effects should be modeled, if they can be properly characterized. Unfortunately, the characterization of the immediate vicinity of nanostructures is very difficult.

In our calculations we have used size corrections to the bulk dielectric function of gold [3]. To understand whether a correction of the bulk dielectric function of gold can effect the predicted optical breakdown thresholds, we calculated maximum near-field enhancement and absorption cross-section for gold nanosphere trimers with different diameters and nanorods with the same aspect ratios. Table 2 shows the relative change in |**E**|_max_ and σ_abs_ when applying the size corrections to the dielectric function of gold. As expected, for large nanostructures (s25t@640 and r25t@930) the optical properties did not change much after including the size-corrected dielectric function of gold. On the other hand, for the smallest nanosphere (s5t@560) and nanorod (r5t@780) |**E**|_max_ and σ_abs_ changed by a factor of 0.17 and 0.40, respectively. In such a cases, not using the size-corrected dielectric function of gold will lead to an underestimation of the optical breakdown threshold.

**Table 2:** The effect of including the size-corrected dielectric function of gold compared to using the bulk dielectric function of gold on the maximum near-field enhancement, (|**E**|_max,bulk_ − |**E**|_max_)/|**E**|_max,bulk_, and absorption cross-section, (σ_abs,bulk_ − σ_abs_)/σ_abs,bulk_ for a gold nanosphere trimer and a gold nanorod at the longitudinal plasmon-resonance wavelength.

nanostructure	(|**E**|_max,bulk_ − |**E**|_max_)/|**E**|_max,bulk_	(σ_abs,bulk_ − σ_abs_)/σ_abs,bulk_

s5t@560	0.17	0.165
s15t@585	0.09	0.066
s25t@640	0.04	0.003
r5t@780	0.40	0.397
r15t@800	0.09	0.044
r25t@930	0.02	0.002

**Figure 3:**
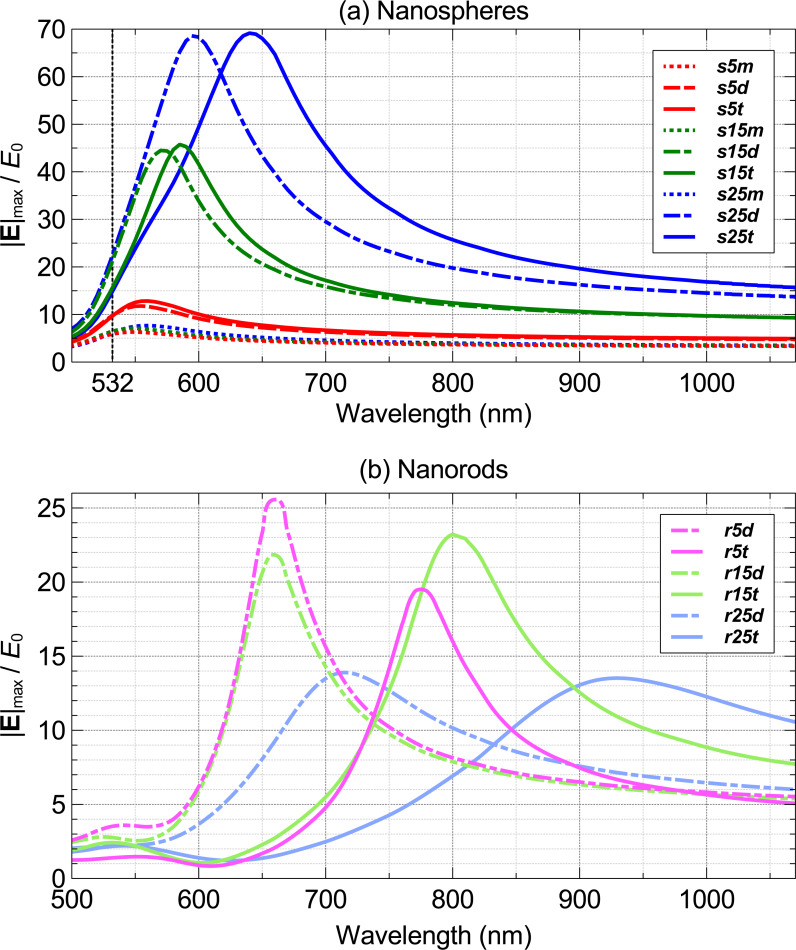
Maximum near-field enhancement, |**E**|_max_/*E*_0_, located at the hot zones of the assemblies or poles of the monomers (see Figure 4a–d). The dimensions of the particles are given in Table 1.

**Figure 4:**
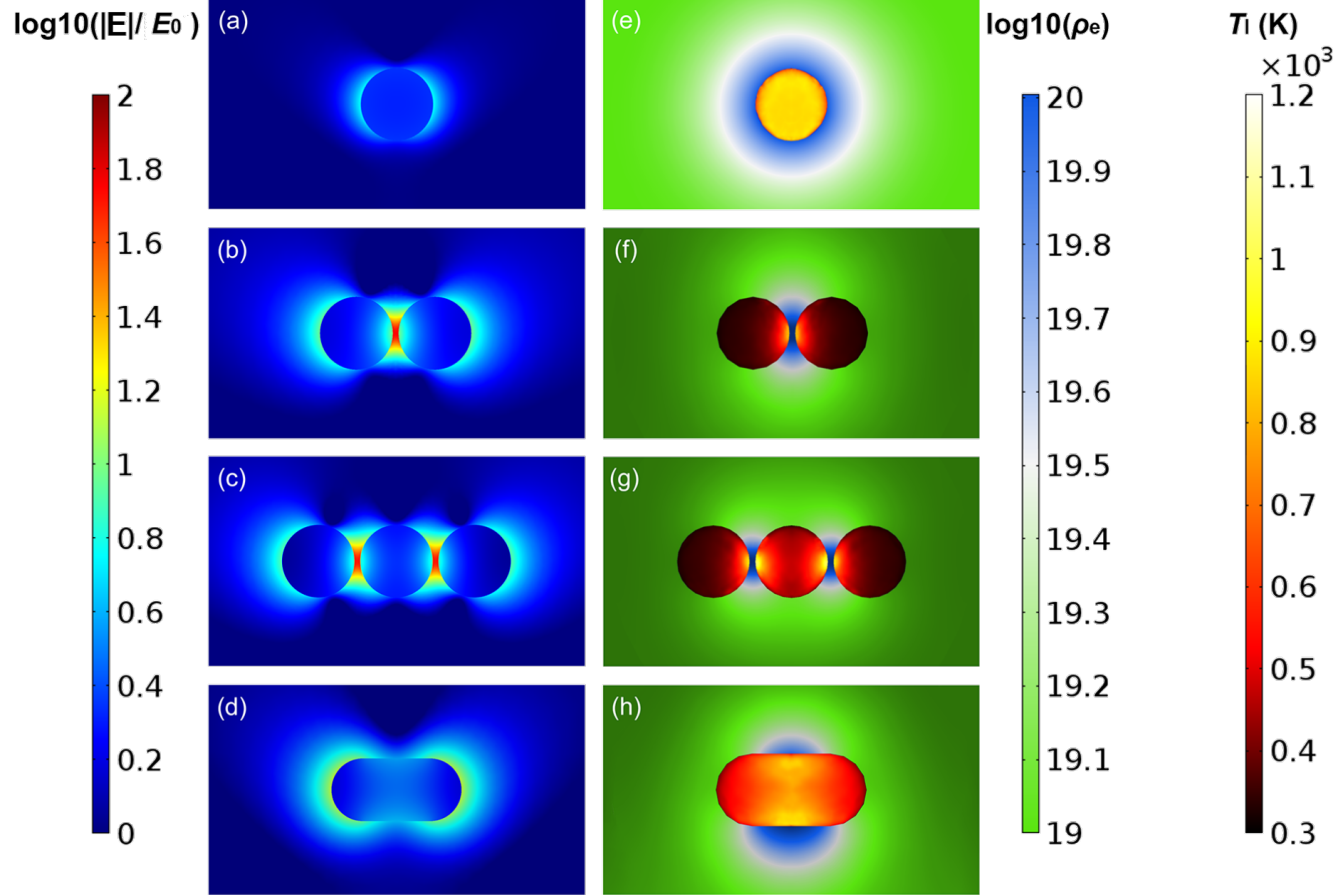
(a–d) Plots of the relative electric field enhancement, log_10_(|**E**|/*E*_0_), of 25 nm nanospheres and nanorods, where |**E**| is the amplitude of the calculated electric field and *E*_0_ is the amplitude of the incident electric field, polarized along the long axis of the nanostructure with propagation from the bottom of the page towards the top. (e–h) the log-scale of free-electron density plasma, log_10_(ρ_e_) (cm^−3^), 4 ps delayed after the temporal peak of the incident laser pulse intensity and the lattice temperature, *T*_l_ (K), of the nanoparticle sampled at the end of the pulse duration. All plots are produced for nanoparticles irradiated at the resonance wavelength (see Figure 5).

**Figure 5:**
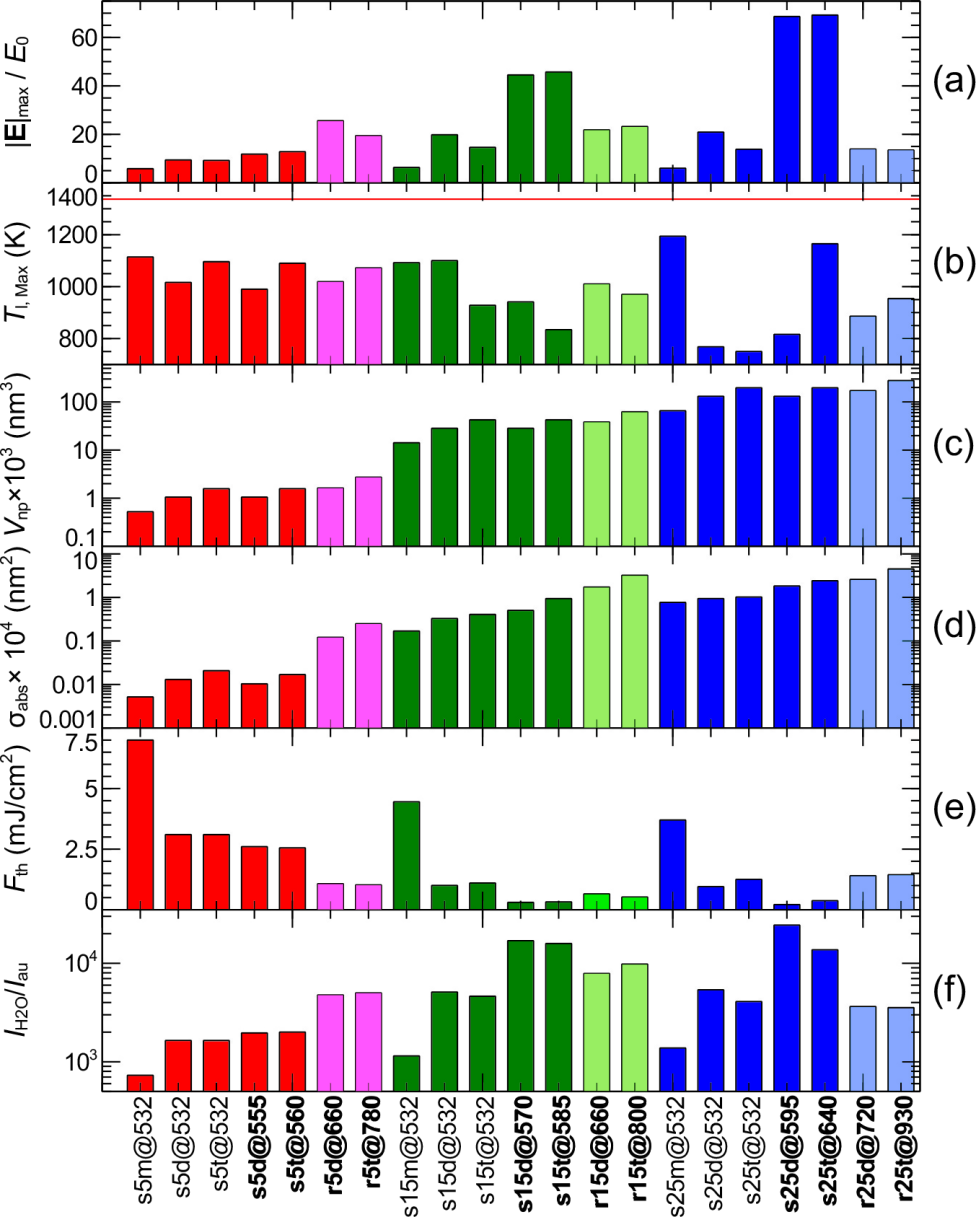
Nanoparticles of different morphology used in the model and corresponding parameters obtained to reach optical breakdown. The first letter of the label (e.g., s5m@532), “s” or “r”, stands for nanosphere and nanorod, respectively. The numbers 5, 15 and 25, correspond to radius of the nanoparticle in nanometers. “m”, “d”, “t” stands for monomer, dimer and trimer, respectively. The last number of the label corresponds to the wavelength, λ (nm), used in the simulations. Bar plot (a), |**E**|_max_/*E*_0_, provides the data of the maximum electric field enhancement that is located in the hot zone of the nanoparticle (see Figure 4a–d). Bar plot (b) shows the maximum lattice temperature, *T*_l,max_, reached at the end of the laser pulse duration, measured in the hot zone of the particle (Figure 4e–h) (the red line marks the melting temperature of gold at 1337 K [38]). Bar plot (c) compares the volumes of the nanoparticles, *V*_np_. Bar plot (d) shows the absorption cross-section, σ_abs_, of the nanoparticles and their assemblies. Bar plot (e) compares the laser fluence, *F*_th_, needed to reach the critical density of the free electrons for bubble formation, ρ_cr_ = 10^20^ cm^−3^ [22,34], in the vicinity of nanoparticle. Bar plot (f) provides the ratio of optical breakdown threshold intensity for pure water for 3 ps pulses at 580 nm, *I*_H2O_ = 8.5 × 10^11^ (W/cm^2^) [56] and an aqueous environment containing gold nanoparticles, *I*_au_ (this study).

The optical properties of the studied nanoparticles presented as spectra of the maximum electric near-field enhancement are given in Figure 3. The position of the maximum of the electric field enhancement for different nanostructures can be seen in Figure 4. For uncoupled nanoparticles (Figure 4a,d) the maximum electric field enhancement is located at the poles of the nanoparticle while for dimers and trimers (Figure 4b,c) the maximum field enhancement is in the region in between the nanoparticles. Figure 5a is an extraction of the maximum near-field enhancement (which we will use throughout this study) from Figure 3, by uncoupled and coupled nanoparticles at on- and off-resonance wavelengths. The wavelength where the maximum near-field enhancement, |**E**|_max_/*E*_0_, peaks is located around λ = 550 nm (Figure 3a), which is close to the second harmonic wavelength of 532 nm of popular solid- state Nd:YAG lasers. |**E**|_max_/*E*_0_ at the peak wavelength for monomers increases with increasing radius, yielding a 2.7% increase from 5 to 25 nm nanospheres (Figure 3a). The change of a nanostructure morphology from a nanosphere monomer to a dimer and trimer induce a red shift in the plasmon resonance peak and leads to an increase of |**E**|_max_/*E*_0_ (Figure 3a). Hovewer, the increase in |**E**|_max_/*E*_0_ at the resonance between a nanosphere dimer and a trimer with the same diameter decreases from 8.5% for 5 nm diameter nanostructures to 0.9% for 25 nm diameter nanostructures (Figure 5a). The highest changes in the maximum near-field enhancement at resonant wavelength were found between the 25 nm nanosphere monomer and its assemblies. The maximum field enhancement of the 25 nm trimer (s25t@640) was 9 times higher than that of the 25 nm monomer at the resonance wavelength and 11.5 times higher than that of the 25 nm monomer (s25m@532) at the second harmonic wavelength of a Nd:YAG laser (532 nm). A plasmon-resonance shift of λ_shift_ = 80 nm can be seen when the assembly builds up from a 25 nm monomer (s25m) to a 25 nm trimer (s25t) (see Figure 3a). For dimer and trimer nanospheres, |**E**|_max_/*E*_0_ increases with the size of a nanosphere and has a small dependence on the number of the nanoparticles. For nanorods, |**E**|_max_/*E*_0_ decreases as the radius of the nanorod increases. The aspect ratio of the nanorods does not affect |**E**|_max_/*E*_0_, except for the 5 nm nanorod (Figure 3b).

Figure 5b shows the maximum lattice temperature, which was sampled in the hot zone of the nanoparticle (Figure 4e–h), *T*_l,max_, of the gold nanoparticles and their assemblies at one full width of the half maximum after the temporal peak of the laser pulse. In the case of the nanosphere monomer, we see almost a uniform distribution of temperature across the nanosphere volume (Figure 4e), while for nanosphere assemblies and the gold nanorod, the temperature profile reveals hot and cold zones across the particle volume (Figure 4f–h). For all nanostructures, the maximum lattice temperature was below the 1337 K, the bulk melting point of gold (Figure 5b), which is still valid for nanoparticles with radii of more than 5 nm [38]. The lowest heating was produced using a gold nanosphere trimer, s25t@532, exposed at the off-resonance wavelength of 532 nm.

The location of the maximum free-electron plasma density surrounding the nanostructures is adjacent to the location of the gold lattice hot temperature spots and to the location of the maximum electric field enhancement inside of the particles (Figure 3b–d and Figure 3f–h). This supports the understanding that during 6 ps pulse interactions gold nanoparticles strongly absorb light (the absorption regime) and photo-thermal emission dominates the production of seed electrons.

Figure 5c,d shows the volume (*V*_np_) and the absorption cross-section (σ_abs_) of the nanoparticles and their assemblies. The absorption cross-section for uncoupled nanoparticles and assemblies increases with the size of the nanoparticle, and also increases when the on-resonance excitation is used. Figure 5e, shows the laser fluence, *F*_th_, needed to reach a critical density of the free electrons, ρ_cr_ = 10^20^ cm^−3^ [22] in the vicinity of the nanoparticle. This corresponds to the free-electron density for bubble formation, which was experimentally observed by Vogel et al. [34]. Although most studies have used a value of ρ_cr_ = 10^21^ cm^−3^ as a critical plasma density required for the optical breakdown [34], we used ρ_cr_ = 10^20^ cm^−3^ due to recent findings by Linz et al. [57–58], which revealed a lower threshold than previously assumed. By comparing Figure 5c, Figure 5d and Figure 5e, we can observe some correlation between *V*_np_, σ_abs_ and *F*_th_, where a higher fluence threshold is needed for smaller absorption cross-sections and volumes of the nanoparticles. Figure 5f, provides the ratio between the optical breakdown threshold intensity for pure water (for 3 ps pulses at 580 nm *I*_H2O_ = 8.5 × 10^11^ W/cm^2^ [56]) and the threshold intensity of water with gold nanoparticles, *I*_au_ (this study). This figure shows that the use of gold nanoparticles and their assemblies can decrease the fluence threshold by up to four orders of magnitude.

**Figure 6:**
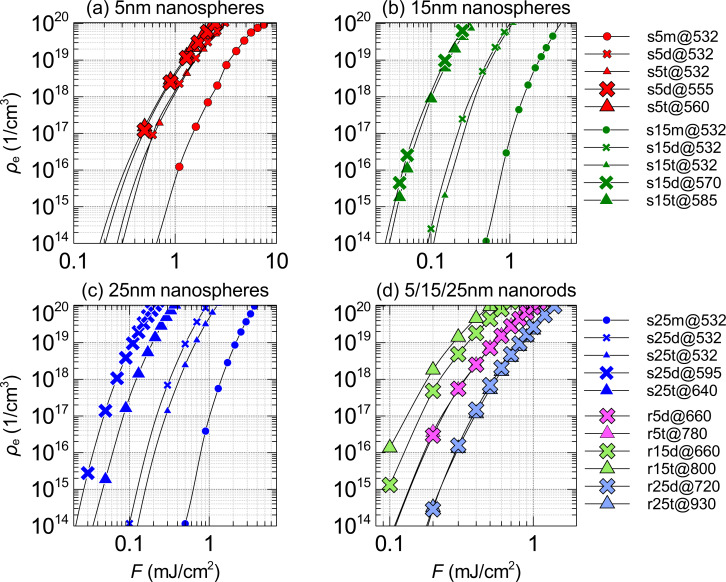
Calculated free-electron densities for different nanoparticle morphologies and wavelengths of 6 ps laser pulses at different fluences, *F*. The legend provides information about nanoparticle type (“s” means sphere and “r” means rod), incident wavelength (e.g., “@532” corresponds to 532 nm) of the laser pulse and the aspect ratio of the nanosphere assembly or nanorod, (“m”: monomer (circles), “d”: dimer (crosses), “t”: trimer (triangles). The numbers 5 (red), 15 (green), 25 (blue) and 5 (light red), 15 (light green), 25 (light blue) corresponds to the radii of nanospheres and nanorods in nanometers, respectively.

Figure 6 shows the impact of the gold nanoparticle morphology (nanosphere monomer, dimer, trimer and nanorods of different size) and laser pulse characteristics (wavelength and fluence) on the plasma density at the location of the highest enhancement of the electric field. A lower fluence is needed to reach the optical breakdown threshold, ρ_cr_, for nanosphere dimers and trimers than for monomers for all three nanoparticle radii. The lowest fluence is needed when nanosphere dimers and trimers are irradiated at their on-resonance wavelengths. In Figure 6d, gold nanorods of the same radius but with different aspect ratios (dimer vs trimer), showed similar laser fluences (see Figure 5e) that are required to achieve the critical plasma density. For example, r5d@660 (nanorod “dimer”) needed *F*_th_ = 1.07 mJ/cm^2^ to reach the critical free-electron density, while r5t@780 (nanorod “trimer”) needed *F*_th_ = 1.02 mJ/cm^2^ to reach the same density.

**Figure 7:**
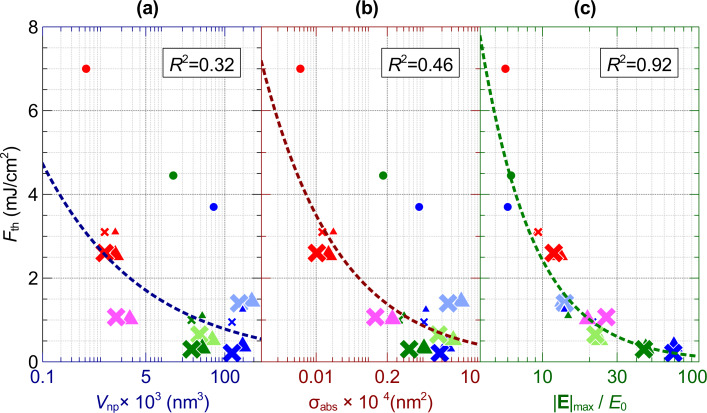
The optical breakdown threshold, *F*_th_, which is required to reach the critical electron density, ρ_cr_ = 10^20^ (cm^−3^), is plotted against (a) the nanoparticle volume, *V*_np_, (b) the absorption cross section, σ_abs_, and (c) the maximum near-field enhancement, |**E**|_max_/*E*_0_. Symbols and color scheme are the same as in Figure 6 at ρ_e_ = 10^20^ cm^−3^.

Figure 7 plots the threshold *F*_th_ as a function of *V*_np_, σ_abs_, |**E**|_max_/*E*_0_. Nanoparticles of different morphology but with comparable volumes need different fluences to reach optical breakdown threshold, which can be seen by comparing r15t@800 against s25m@532; s15t@585, r15d@660 and s15t@532 against each other; and r5d@660 s5t@560 and s5t@532 against each other. On the other hand, an on-resonance irradiated s25t@640 and r25d@720 with comparable absorption cross-sections had *F*_th_ = 0.37 mJ/cm^3^ and *F*_th_ = 1.4 mJ/cm^3^, respectively. A similar situation can be seen comparing s25d@595 against r15d@660, and s15t@585, s25d@532 and s25t@532 against each other, where the nanoparticles with different morphology but comparable absorption cross-section have different optical breakdown thresholds. A power regression fit of the optical breakdown threshold versus the volume of the nanoparticle yielded, *F*_th_ = 2.5969*V*_np_^−0.26^ with *R*^2^ = 0.32, and a power regression fit of the optical breakdown threshold versus the absorption cross-section yield, *F*_th_ = 0.8363σ_abs_^−0.311^ with *R*^2^ = 0.46. Figure 7c shows the relation between the maximum field enhancement for different morphologies, wavelengths of laser irradiation and optical breakdown threshold. A power regression fit of the optical breakdown versus the maximum field enhancement yielded, *F*_th_ = 44.96(|**E**|_max_/*E*_0_)^−1.267^ with *R*^2^ = 0.92. The strong dependence of the optical breakdown threshold on the near-field enhancement compared to the poor dependence on morphology and absorption cross-section is important knowledge for the design of appropriate nanostructures for lowering the optical breakdown threshold.

## Conclusion

We have shown that the optical breakdown threshold for picosecond-pulse interaction with gold nanoparticles of different morphologies is highly dependent on the near-field enhancement in the vicinity of the nanoparticle and to a lesser degree on type, volume and absorption cross-section of the nanoparticle. In the case of uncoupled nanoparticles the optical breakdown threshold is highly dependent on both the absorption cross-section and the near-field enhancement by the nanoparticle due to the similarity in their spectral shapes. The results obtained show that the use of nano-assemblies can lower the threshold by four orders of magnitude in comparison to pure water [56]. These findings can further advance the use of gold nanoparticles and their assemblies for applications, such as gold-mediated transfection and opto-poration [45,59–61], nanoparticle-enhanced laser-induced breakdown spectroscopy [20–21], cell nanosurgery [19], drug release [62–63], fabrication of functional gold-antibody nanoconjugates [64] and imaging [65].

## Supporting Information

File 1 Theory and parameters used in the model.
