# Supplementary material for: The role of morphology and coupling of gold nanoparticles in optical breakdown during picosecond pulse exposures
Source: Beilstein J Nanotechnol. 2016 Jun 16;7:869–80. doi: 10.3762/bjnano.7.79 (PMC4979631; doi:10.3762/bjnano.7.79)
Supplement: File 1 — Theory and parameters used in the model. [file Beilstein_J_Nanotechnol-07-869-s001.pdf]

Supporting information for: The role of gold nanoparticle morphology and coupling on optical breakdown during picosecond pulse exposures

Yevgeniy R. Davletshin and J. Carl Kumaradas*

Department of Physics, Ryerson University, Toronto

E-mail: ckumarad@ryerson.ca

Phone: +1 416-979-5000 x7462. Fax: +1 416-979-5343

Theory and parameters used in the model

Electromagnetic field interaction

Several physical phenomena need to be fully coupled in order to fully characterize a low density plasma formation in the vicinity of gold nanoparticles and their assemblies in aqueous medium. The electric field, $\mathbf{E}$, produced in and around gold nanoparticles was divided into incident electric field, $\mathbf{E}_{\text{inc}}$, and scattered field, $\mathbf{E}_{\text{sca}}$: $\mathbf{E} = \mathbf{E}_{\text{inc}} + \mathbf{E}_{\text{sca}}$. $\mathbf{E}_{\text{inc}}$ was imposed on the domain boundary and $\mathbf{E}_{\text{sca}}$ was calculated in the domain using Helmholtz wave equation (details on the individual symbols and constants used in the theory and model can be find in the Table S1):

$$\nabla \times \nabla \times \mathbf{E}_{\text{sca}} - \omega^2 \varepsilon_0 \mu_0 \tilde{\varepsilon} \mathbf{E}_{\text{sca}} = 0, \tag{1}$$

where $\tilde{\varepsilon}$, is the size-dependent dielectric constant for gold. It is given by:[S1]

$$\tilde{\epsilon}(\omega, L_{\text{eff}}) = \tilde{\epsilon}_{\text{bulk}} + \frac{\omega_p^2}{\omega^2 + i\omega\gamma_0} - \frac{\omega_p^2}{\omega^2 + i\omega\left(\gamma_0 + \frac{Av_{\text{F}}}{L_{\text{eff}}} + \frac{\eta V_{\text{au}}}{\pi}\right)}. \tag{2}$$

The incident light was polarized along the y-axis (the longest axis of the nanostructure) and travelled along the positive z-direction. Its electric field was, $\mathbf{E}_{\text{inc}}$:

$$\mathbf{E}_{\text{inc}} = \vec{y}E_0 \cdot e^{-j \cdot \kappa_{\text{w}} \cdot z}, \tag{3}$$

where κ_{w} is the wave number in medium. The dielectric function of water was modelled using Drude formalism, to account for a presents of free electron plasma as:

$$\varepsilon_{\text{w}} = \varepsilon_{\infty} - \frac{ne^2}{\varepsilon_0 m(\omega^2 + j\omega/\tau)}, \tag{4}$$

where ε_{∞} is the water relative permittivity (assuming biologically relative refractive index of 1.4), m is the electron reduced mass and τ is the mean free time between electron/molecule collisions.

The size-dependent corrections to the complex dielectric function of gold, $\tilde{\varepsilon}$,are based on the recent quantitative comparison of a finite element model of a single gold nanorod against spatial modulation spectroscopy (SMS) technique.[S1] The solution to the Helmholtz wave equation provides the optical behaviour of a gold nanoparticle, such as electric field enhancement, $|\mathbf{E}|/E_0$ and absorption cross-section, σ_{abs}. The combination of perfect matched layer (PML) domain and absorbing boundary condition was used to truncate electromagnetic simulations domain and reduce reflections from artificial boundaries. The perfect electric (PEC) and perfect magnetic (PMC) conductor boundaries were used to truncate domain to one quarter of full 3D geometry.

Two temperature model

The resistive losses during laser pulse interaction with gold nanoparticle, Q_{rh}, calculated from the electromagnetic model is fed into a hyperbolic two-temperature model (TTM)[S2] to solve for the energy transfer between the laser pulse and the conduction electrons of gold due to electron-phonon relaxation and heat diffusion from the gold lattice to the surrounding medium through interface conductance, $Q_{\mathrm{au|w}}$:[S3]

$$C_{\mathrm{e}}\frac{\partial T_{\mathrm{e}}}{\partial t} + \nabla \cdot \boldsymbol{q}_{\mathrm{e}} = -G(T_{\mathrm{e}} - T_{\mathrm{l}}) + Q_{\mathrm{rh}} \cdot f(\tau_{\mathrm{w}}), \tag{5}$$

$$\tau_{\mathrm{e}}\frac{\partial \boldsymbol{q}_{\mathrm{e}}}{\partial t} + \boldsymbol{q}_{\mathrm{e}} = -\kappa_{\mathrm{e}}\nabla T_{\mathrm{e}} \tag{6}$$

$$C_{\mathrm{l}}\frac{\partial T_{\mathrm{l}}}{\partial t} + \nabla \cdot \boldsymbol{q}_{\mathrm{l}} = G(T_{\mathrm{e}} - T_{\mathrm{l}}), \tag{7}$$

$$\tau_{\mathrm{l}}\partial\frac{\boldsymbol{q}_{\mathrm{l}}}{\partial t} + \boldsymbol{q}_{\mathrm{l}} = -\kappa_{\mathrm{l}}\nabla T_{\mathrm{l}}, \tag{8}$$

$$Q_{\mathrm{au|w}} = q_0(T_{\mathrm{l}} - T_{\mathrm{m}}). \tag{9}$$

Plasma formation

The temperature rise of the conduction electrons in gold from the TTM and the electric field distribution from the electromagnetic model are used as input parameters to the plasma model. The interaction of the strong electromagnetic fields with the electrons in a aqueous medium can lead to multiphoton, impact and avalanche ionization of the electrons into a quasi-free state in the conduction band. When the density of the excited electrons reaches a critical value of $10^{18}-10^{20}$ cm^{-3},[S4–S9] the electron cloud starts to gain sufficient kinetic energy from the interaction with the laser pulse that an optical breakdown occurs. Laser-induced

breakdown can lead to the breakage of atomic bounds, bubble formation, luminescence and acoustic shock formation. In order to determine threshold of plasma induced optical breakdown in aqueous media, the medium can be represented as water[S10,S11] with properties of an amorphous semiconductor with a band gap energy of $E_{\mathrm{gap}} = 6.5$ eV.[S7,S12,S13] To model the changes in free electron density and determine the irradiance threshold for optical breakdown, the generic form of the rate equation for free electron density, ρ_{e}, can be used:[S11]

$$\frac{d\rho_{\mathrm{e}}}{dt} = \left(\frac{d\rho_{\mathrm{e}}}{dt}\right)_{\mathrm{photo}} + \left(\frac{d\rho_{\mathrm{e}}}{dt}\right)_{\mathrm{casc}} + \left(\frac{d\rho_{\mathrm{e}}}{dt}\right)_{\mathrm{diff}} + \left(\frac{d\rho_{\mathrm{e}}}{dt}\right)_{\mathrm{rec}} \tag{10}$$

The $\left(\frac{d\rho_{\mathrm{e}}}{dt}\right)_{\mathrm{photo}}$ term on the right-hand side of Equation (10), models photoionization of electrons via multiphoton absorption and tunnel ionization.[S14–S16] The $\left(\frac{d\rho_{\mathrm{e}}}{dt}\right)_{\mathrm{casc}}$ term adds the contribution of cascade ionization (sometimes called avalanche ionization) via inverse Bremsstrahlung absorption.[S17–S19] The $\left(\frac{d\rho_{\mathrm{e}}}{dt}\right)_{\mathrm{diff}}$ and $\left(\frac{d\rho_{\mathrm{e}}}{dt}\right)_{\mathrm{rec}}$ terms represent free electron plasma diffusion and recombination, respectively.

To complete the picture of the free electron plasma formation by laser pulse interactions with a gold nanoparticle, two additional processes needs to be considered: thermal ionization (thermionic emission),[S20,S21] ρ_{therm}, and photo-thermal emission,[S22] ρ_{au}.

The thermal ionization starts to play a significant role in free electron production when the temperature in the focal volume of the laser is sufficiently high (≈ 5000 K and above[S20,S21]) and large free electron densities are already achieved by multiphoton and cascade ionization. On the other hand, thermal ionization also plays a role during sequences of ultrashort laser pulses, when the time delay between pulses is in the order of thermalization time of the electron plasma energy.[S21] Thermal ionization also partially depletes the density of bound electrons in the valence band and reduces the rate of multiphoton and impact ionization.[S20] Although in the model, the temperature of the medium during single pulse illumination is sufficiently lower than 5000 K, we incorporated thermal ionization therm, based on derivations given by Linz *et al.*,[S20] into free electron density rate Equation (10) to have complete picture of the free electron plasma generation, so that it can be used for future studies of

ultrashort pulses of a high repetition rates. Please note that in present single 6 ps pulse study thermal ionization of the water is negligible and can be omitted.

Photo-thermal emission of hot electrons on the boundary of the gold, also starts to play a role when the temperature of the nanoparticle's electrons rises to a level where the electrons can cross the metal/medium energetic barrier of $W_{\mathrm{au}} = 3.72$ eV and contribute to electron density plasma formation.[S23]

4 terms in the generic rate Equation (10), plus thermal ionization of water, ρ_{therm}, and photo-thermal emitted electron current density across gold/water boundary, of low density plasma formation are expanded as follows:

The photoionization rate,[S20] $\left(\frac{d\rho_{\mathrm{e}}}{dt}\right)_{\mathrm{photo}}$:

$$\left(\frac{d\rho_{\mathrm{e}}}{dt}\right)_{\mathrm{photo}} = \frac{2\omega}{9\pi}\left(\frac{m\omega\sqrt{1+\gamma^2}}{\hbar\gamma}\right)^{3/2} \mathrm{Q}\left(\gamma, \frac{\tilde{\Delta}}{\hbar\omega}\right) \times \left(\frac{\rho_{\mathrm{bound}} - \rho_{\mathrm{e}} + \rho_{\mathrm{au}}}{\rho_{\mathrm{bound}}}\right)$$
$$\exp\left\{-\pi\left\langle -\frac{\tilde{\Delta}}{\hbar\omega} + 1\right\rangle \times \left[\mathcal{K}\left(\frac{\gamma}{\sqrt{1+\gamma^2}}\right) - \mathcal{E}\left(\frac{\gamma}{\sqrt{1+\gamma^2}}\right)\right] \Big/ \mathcal{E}\left(\frac{1}{\sqrt{1+\gamma^2}}\right)\right\};$$

$$\mathrm{Q}(\gamma, \mathrm{x}) = \sqrt{\frac{\pi}{2\mathcal{K}\left(\frac{1}{\sqrt{1+\gamma^2}}\right)}} \times \sum_{l=0}^{\infty} \exp\left\{-\pi l\left[\mathcal{K}\left(\frac{\gamma}{\sqrt{1+\gamma^2}}\right) - \mathcal{E}\left(\frac{\gamma}{\sqrt{1+\gamma^2}}\right)\right] \Big/ \mathcal{E}\left(\frac{1}{\sqrt{1+\gamma^2}}\right)\right\}$$
$$\times\Phi\left\{\left[\pi^2(2\langle x+1\rangle - 2x + l)\Big/ 2\mathcal{K}\left(\frac{1}{\sqrt{1+\gamma^2}}\right)\mathcal{E}\left(\frac{1}{\sqrt{1+\gamma^2}}\right)\right]^{1/2}\right\}, \tag{11}$$

where γ is the Keldysh parameter given by:

$$\gamma = \frac{\omega}{\omega_{\mathrm{t}}} = \omega\frac{\sqrt{mE_{\mathrm{gap}}}}{e\mathbf{E}} = \frac{\omega}{e}\sqrt{\frac{c\epsilon_0 m E_{\mathrm{gap}} n}{2\mathbf{I}_{\mathrm{tot}}(t)}}; \tag{12}$$

here, $1/\omega_{\mathrm{t}}$, is the tunnelling time through the atomic potential barrier, m is the electron reduced mass, and $\tilde{\Delta}$ is the effective ionization potential for creating an electron-hole pair

in a condensed matter exhibiting a band structure and corrected for the oscillation energy of the electron due to the electromagnetic field. It is given by:

$$\tilde{\Delta} = \frac{2}{\pi} E_{\mathrm{gap}} \frac{\sqrt{1+\gamma^2}}{\gamma} \mathcal{E}\left(\frac{1}{\sqrt{1+\gamma^2}}\right). \tag{13}$$

In Equation (11), $< x >$ represents the integer part of the number x, $\mathcal{K}()$ and $\mathcal{E}()$ denote the elliptic integrals of the first and second kind, and $\Phi()$ denotes the Dawson probability integral:

$$\Phi(z) = \int_0^z \exp{(y^2 - z^2)} dy. \tag{14}$$

The cascade ionization rate,[S20] $\left(\frac{d\rho_{\mathrm{e}}}{dt}\right)_{\mathrm{casc}}$

Cascade ionization contributes to the rate Equation (10) only after certain initial free electron density, ρ_{seed}, has been reached by either photoionization or photo-thermal emission. The initial seed free electron density is the fitting parameter and is chosen based on 50 % probability of having at least one seed electron in the focal volume of laser.[S20] Then cascade ionization rate can be written as:

$$\left(\frac{d\rho_{\mathrm{e}}}{dt}\right)_{\mathrm{casc}} = \begin{cases} \frac{\alpha_{\mathrm{casc}} I_{\mathrm{inc}}(t)}{1+\eta_{\mathrm{casc}} t_{\mathrm{ret}}} \times \rho_{\mathrm{e}} - \frac{\beta_{\mathrm{casc}}}{1+\eta_{\mathrm{casc}} t_{\mathrm{ret}}} \times (\rho_{\mathrm{e}} - \rho_{\mathrm{therm}}) & \text{for } \rho_{\mathrm{e}} \geq \rho_{\mathrm{seed}} \\ 0 & \text{for } \rho_{\mathrm{e}} < \rho_{\mathrm{seed}} \end{cases} \tag{15}$$

Thermal ionization,[S20] ρ_{therm}

Based on derivation of Linz *et al.*,[S20] the third process of free electron generation can come from thermalization of energy, carried by primary free electrons via thermal emission. Thermally ionized free electron density in the conduction band is calculated by:

$$\left(\frac{\partial\rho_{\mathrm{therm}}}{\partial t}\right)=\frac{\partial T_{\mathrm{w}}}{\partial t}\left(\frac{3\kappa_{\mathrm{b}}}{E_{\mathrm{gap}}}+\frac{1}{T_{\mathrm{w}}}\right)\frac{3}{2}\sqrt{\frac{\pi}{2}}\left(\frac{\kappa_{\mathrm{b}}T_{\mathrm{w}}}{E_{\mathrm{gap}}}\right)^{\frac{1}{2}}exp\left(-\frac{E_{\mathrm{gap}}}{2\kappa_{\mathrm{b}}T_{\mathrm{w}}}\right)(\rho_{\mathrm{bound}}-\rho_{\mathrm{e}}) \tag{16}$$

Photo-thermal emission current, $\boldsymbol{J}_{\mathrm{au}}$

The photo-thermal emitted electron current density across metal/medium boundary can be described by generalized Fowler-DuBridge theory of multiphoton photoemission at high temperatures:[S24,S25]

$$\boldsymbol{J}_{\mathrm{au}}=A_0T_{\mathrm{e}}^2\exp(-\frac{\mathrm{eW_{au}}}{\kappa_{\mathrm{b}}T_{\mathrm{e}}})+c_{\mathrm{au}}\frac{2(\kappa_{\mathrm{b}}T_{\mathrm{e}})^2}{4(3\hbar\omega-W_{\mathrm{au}})^2}F(\frac{(3\hbar\omega-W_{\mathrm{au}})}{\kappa_{\mathrm{b}}T_{\mathrm{e}}})(1-R)^3\boldsymbol{I}_{\mathrm{tot}}^3(t)\mathbf{n} \tag{17}$$

where A_0 is the Richardson coefficient (120 A/cm^2/K^2), W_{au} is work function (3.72 eV),[S23] F is the so called Fowler function,[S24,S25] c_{au} is the three-photon ionization cross-section (1×10^{-7} Acm^4/MW^3),[S26] κ_{b} is the Boltzmann constant and R is the reflection coefficient of the gold. The photo-thermal electron current density is set as a boundary flux/source condition across gold/water interface.

The details for diffusion, $\left(\frac{d\rho_{\mathrm{c}}}{dt}\right)_{\mathrm{diff}}$, and electron-hole recombination rates, (η_{rec}) of the Equation (10) are given in the Table S1. The first one is based on the characteristic diffusion length, Λ, which is set to the radius of the gold nanoparticle.[S27] The electron-hole recombination rate is set to empirical value obtained by Docchio.[S28]

By implementing above mentioned terms into generic rate Equation (10), plus introducing photo-thermal emission current as a boundary condition, leads to a full description of free electron plasma formation in the vicinity of gold nanoparticle in the form:

$$\frac{d\rho_{\mathrm{e}}}{dt}=\left(\frac{d\rho_{\mathrm{e}}}{dt}\right)_{\mathrm{photo}}+\left(\frac{d\rho_{\mathrm{e}}}{dt}\right)_{\mathrm{casc}}+\left(\frac{d\rho_{\mathrm{e}}}{dt}\right)_{\mathrm{diff}}+\left(\frac{d\rho_{\mathrm{e}}}{dt}\right)_{\mathrm{rec}}+\left(\frac{d\rho_{\mathrm{therm}}}{dt}\right). \tag{18}$$

The transient changes in the optical behaviour of the s25t@640 nanoparticle with a fluence of 0.37 mJ/cm^2 are shown in Figure S1. The dark red line in Figure S1 represents the tempo-

ral evolution of maximum electric field enhancement in the middle of 4 nm gap between two adjacent nanospheres. As the density of free electron plasma, ρ_e, reaches 1×10^{19} cm^{-3} the plasma becomes highly absorbent to incident laser irradiation and shields the gold nanoparticle. The maximum field enhancement reaches its minimum at the maximum density of the plasma (dip in the near-field enhancement, Figure S1). The 4 ps difference between peak of the laser intensity and the peak of the free electron density is caused by a domination of photo-thermal emission of hot electrons off the nanoparticle's surface, where photo-thermal emission is proportional to $T_e^2 \mathbf{I}_{tot}^3$.

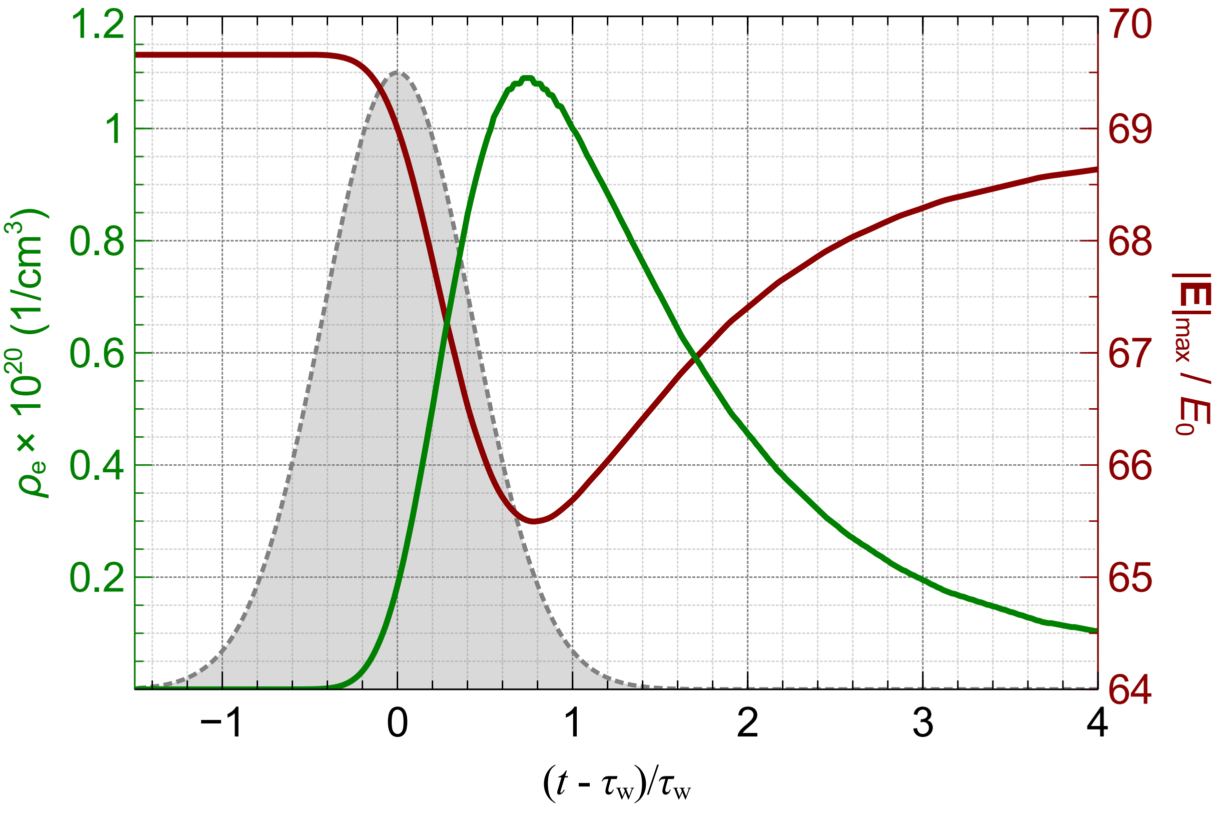

Figure S1: Temporal evaluation free electron plasma, ρ_e (cm^{-3}), shown by the green line; and maximum electric field enhancement, $|\mathbf{E}|_{max}/E_0$, shown by the dark red line for 25 nm nanosphere trimer (s25t@640) irradiated at the resonance peak wavelength, 640 nm, with the fluence of 0.37 mJ/cm^3. The probe point located at the middle of the edge-to-edge gap between two adjacent nanoparticles. The grey shaded area shows the laser intensity profile (in arbitrary units). The time is normalized to FWHM of the laser pulse, τ_w.

Heat transfer in aqueous media

The temperature increase in the aqueous media, T_{w}, due to the laser-particle interaction, linear absorption (Joule heating), plasma formation and optical breakdown, is modelled by solving heat transfer equation. There were four types of heat sources: 1) a Dirichlet boundary condition of the interface conductance at the nanoparticle surface - Equation (9); 2) Joule heating with the plasma produced in the vicinity of the nanoparticle, Q_{rh}; 3) electron collision losses with neutral molecules during impact ionization, $\left(\frac{dT_{\mathrm{m}}}{dt}\right)_{\mathrm{coll}}$; 4) heating through electron recombination of ionized molecules, $\left(\frac{dT_{\mathrm{m}}}{dt}\right)_{\mathrm{rec}}$. With above mentioned considerations, the heat transfer equation will following:

$$\rho_{\mathrm{w}} c_{\mathrm{w}} \frac{\partial T_{\mathrm{w}}}{\partial t} = \nabla(\kappa_{\mathrm{w}} \nabla T_{\mathrm{w}}) + Q, \tag{19}$$

where heat source, Q, is:

$$Q = Q_{\mathrm{rh}} \cdot f(\tau_{\mathrm{w}}) + \left(\frac{dT_{\mathrm{m}}}{dt}\right)_{\mathrm{coll}} + \left(\frac{dT_{\mathrm{m}}}{dt}\right)_{\mathrm{rec}}. \tag{20}$$

Table S1: Symbols and constants used in the theory

Symbol	Value/Expression	Description
λ		Laser wavelength
c_0	299792458 m/s	Speed of light in vacuum
ω	$\frac{2\pi c_0}{\lambda}$	Angular frequency
ε_0	8.854 187 817... $\times 10^{-12}$ F/m	Vacuum permittivity
$\tilde{\varepsilon}$	eq. 2	Size-dependant dielectric constant [S1]
$\mathbf{E}$	eq. 1	Electric field strength
E_0	$\sqrt{\frac{2I_{\mathrm{inc}}}{c_0 n \varepsilon_0}}$	Incident electric field
$f(\tau_{\mathrm{w}})$	$\frac{2.35482}{\tau_{\mathrm{w}}\sqrt{2\pi}} e^{\frac{-(t-\tau_{\mathrm{w}})^2}{2(2.35482)^2}}$	Gaussian shape pulse function
τ_{w}	6 ps	Laser pulse width [full width at half maximum (FWHM)]
I_{inc}	$\frac{c_0 n \varepsilon_0 \lvert E_0^2 \rvert}{2}$	Incident irradiance of laser light
e	1.602176565(35)$\times 10^{19}$ C	Electron charge

Continued on next page

Table S1 – *Continued from previous page*

**Symbol**	**Value/Expression**	**Description**
ρ_e	see eq. 10	Density of free electron plasma
m_e	$9.10938291(40)\times10^{31}$ kg	Electron mass
$\tilde{\epsilon}_\mathrm{bulk}$	see Johnson and Christy[S29]	Bulk dielectric function
μ_0	$4\pi \times 10^{7}$ N / A^2	Magnetic permeability in vacuum
ω_p	$\sqrt{\frac{\rho_\mathrm{e} e^2}{\varepsilon_0 m_\mathrm{e}}}$	Plasma frequency of free electron gas
γ_0	1.094×10^{14} 1/s	Bulk relaxation frequency[S29]
v_F	1.41 nm/fs	Fermi velocity[S30]
L_eff	$\frac{4V_\mathrm{au}}{S_\mathrm{au}}$	Reduced effective mean free path length[S31]
V_au		Volume of the particle
S_au		Surface area of the particle
η	$(5.5 \pm 1.5) \times 10^{-7}$ fs^{-1} nm^{-3}	Radiation damping proportionality constant[S32]
A	0.5	Broadening parameter[S1]
σ_abs	$\frac{1}{I_\mathrm{inc}} \int_{V_\mathrm{au}} \Re(Q_\mathrm{rh}) dV_\mathrm{au}$	Absorption cross-section of the gold nanoparticle[S33]
$\Re$		Real part
n	1.40	Refractive index of water[S34]
$\mathbf{D}$		Dielectric displacement
σ		Electric conductivity
$\Im$		Imaginary part
Q_rh	$\frac{1}{2}\Re[(\sigma - j\omega\varepsilon)\mathbf{E}\cdot\mathbf{E}^*]$	Resistive losses during laser pulse interaction[S33]
C_e	see ref.[S3]	Electronic heat capacity of gold
C_l	see ref.[S3]	Lattice heat capacity of gold
T_e	see eq. 5	Electronic temperature of gold
T_l	see eq. 7	Lattice temperature of gold
$\boldsymbol{q}_\mathrm{e}$	see eq. 6	Electronic heat flux vector (TTM model)
$\boldsymbol{q}_\mathrm{l}$	see eq. 8	Lattice heat flux vector (TTM model)
G	2×10^{16} Wm^{-3}K^{-1}	Electron-phonon coupling factor[S3]
τ_e	$(1.2 \times 10^{7} * T_\mathrm{e}^2 + 1.23 \times 10^{11} * T_\mathrm{l})^{-1}$ s	Electron collision time[S2]
τ_l	0.8 ps	Ion collision time[S35]
κ_e	see ref.[S35]	Electron thermal conductivity
κ_l	see ref.[S22]	Lattice thermal conductivity

Continued on next page

**Symbol**	**Value/Expression**	**Description**
$\rho_{\rm bound}$	$6.68 \times 10^{22}\ e/{\rm cm}^3$	Bound electron density of water[S36]
$\mathbf{I}_{\rm tot}$	$\frac{c_0 n \varepsilon_0 \mathbf{E}^2}{2} f(\tau_{\rm w})$	Irradiance of laser pulse
$\eta_{\rm casc}$	$\frac{1}{\omega^2\tau^2+1}\left[\frac{{\rm e}^2\tau}{c_0 n \varepsilon_0 m_{\rm e}(3/2)\tilde{\Delta}}\mathbf{I}_{\rm tot}(t) - \frac{m_{\rm e}\omega^2\tau}{M}\right]$	Cascade ionization rate[S36]
$\tilde{\Delta}$	see eq. 13	Effective ionization potential
γ	see eq. 12	Keldysh parameter[S14,S20]
M	3×10^{-26} kg	Mass of water molecule
τ	1.6 fs	Mean free time between electron/molecule collisions[S20]
$t_{\rm ret}$	$\tau \left\langle 1 + \frac{3\tilde{\Delta}}{2\hbar\omega} \right\rangle$	Cascade retardation time[S20]
$\hbar$	$1.98644568{\times}10^{-25}$ J·m	Reduced Planck constant
$E_{\rm gap}$	6.5 eV	Band gap energy of water[S7,S12]
$\alpha_{\rm casc}$	$\frac{1}{\omega^2\tau^2+1}\frac{2{\rm e}^2\tau}{3cn\epsilon_0 m_{\rm e}E_{\rm gap}}\left(1 - \frac{\rho_{\rm e}+\rho_{\rm au}}{\rho_{\rm bound}}\right)$	Gain in cascade ionization[S20]
$\beta_{\rm casc}$	$\frac{m_{\rm e}}{M}\frac{\omega^2\tau}{\omega^2\tau^2+1}\left(1 - \frac{\rho_{\rm e}+\rho_{\rm au}}{\rho_{\rm bound}}\right)$	Collision loss tern of cascade ionization[S20]
$\left(\frac{d\rho_{\rm e}}{dt}\right)_{\rm diff}$	$\frac{\tau(5/4)\tilde{\Delta}}{3m_{\rm e}\Lambda^2}\cdot(\rho_{\rm e} - \rho_{\rm therm})$	Diffusion rate[S27]
Λ	set as a radius of gold nanoparticle	Characteristic diffusion length[S27]
$\eta_{\rm rec}$	$2 \times 10^{-9}{\rm cm}^3/{\rm s}$	Empirical recombination rate[S28]
$\left(\frac{d\rho_{\rm e}}{dt}\right)_{\rm rec}$	$-\eta_{\rm rec}\cdot(\rho_{\rm e} - \rho_{\rm therm})^2$	Recombination of free electron plasma
$\left(\frac{dT_{\rm m}}{dt}\right)_{\rm coll}$	$\frac{5}{4c_{\rm w}\rho_{\rm w}}E_{\rm gap}\beta_{\rm casc}(\rho_{\rm e} - \rho_{\rm therm})$	Heating through collision losses during impact ionization[S20]
$\left(\frac{dT_{\rm m}}{dt}\right)_{\rm rec}$	$\frac{5}{4c_{\rm w}\rho_{\rm w}}E_{\rm gap}\eta_{\rm rec}(\rho_{\rm e} - \rho_{\rm therm})^2$	Heating through recombination[S20]
$\rho_{\rm au}$	see eq. 18	Density of thermally ionized electrons in the medium[S20,S21]
$\rho_{\rm therm}$	see eq. 17	Density of electrons emitted off the gold surface[S25]
$c_{\rm w}$	4184 J/kg/K	Heat capacity of water
$\rho_{\rm w}$	$1000\ {\rm kg/m}^3$	Density of water
$\kappa_{\rm w}$	0.61 W/m/K	Thermal conductivity of water
$T_{\rm w}$	see eq. 19	Temperature of water
q_0	$105.0 \times 10^6\ {\rm W/m}^2/K$	Thermal conductance at gold-water interface[S37]

References

(S1) Davletshin, Y. R.; Lombardi, A.; Cardinal, M. F.; Juvé, V.; Crut, A.; Maioli, P.; Liz-Marzán, L. M.; Vallée, F.; Del Fatti, N.; Kumaradas, J. C. A Quantitative Study of the Environmental Effects on the Optical Response of Gold Nanorods. *ACS Nano* **2012**, *6*, 8183–8193.

(S2) Chen, J.; Tzou, D.; Beraun, J. A semiclassical two-temperature model for ultrafast laser heating. *Int. J. Heat Mass Transf.* **2006**, *49*, 307–316.

(S3) Ekici, O.; Harrison, R. K.; Durr, N. J.; Eversole, D. S.; Lee, M.; Ben-Yakar, A. Thermal analysis of gold nanorods heated with femtosecond laser pulses. *J. Phys. D. Appl. Phys.* **2008**, *41*, 185501.

(S4) Vogel, A.; Nahen, K.; Theisen, D.; Noack, J. Plasma formation in water by picosecond and nanosecond Nd:YAG laser pulses. I. Optical breakdown at threshold and superthreshold irradiance. *IEEE J. Sel. Top. Quantum Electron.* **1996**, *2*, 847–860.

(S5) Noack, J.; Vogel, A. Laser-induced plasma formation in water at nanosecond to femtosecond time scales: calculation of thresholds, absorption coefficients, and energy density. *IEEE J. Quantum Electron.* **1999**, *35*, 1156–1167.

(S6) Kennedy, P. A first-order model for computation of laser-induced breakdown thresholds in ocular and aqueous media. I. Theory. *Quantum Electron., IEEE J.* **1995**, *31*, 2241–2249.

(S7) Sacchi, C. A. Laser-induced electric breakdown in water. *J. Opt. Soc. Am. B* **1991**, *8*, 337.

(S8) Bloembergen, N. Laser-induced electric breakdown in solids. *Quantum Electron., IEEE J.* **1974**, *10*, 375–386.

(S9) Feng, Q.; Moloney, J.; Newell, A.; Wright, E.; Cook, K.; Kennedy, P.; Hammer, D.; Rockwell, B.; Thompson, C. Theory and simulation on the threshold of water breakdown induced by focused ultrashort laser pulses. *Quantum Electron., IEEE J.* **1997**, *33*, 127–137.

(S10) Venugopalan, V.; Guerra, A.; Nahen, K.; Vogel, A. Role of Laser-Induced Plasma Formation in Pulsed Cellular Microsurgery and Micromanipulation. *Phys. Rev. Lett.* **2002**, *88*, 078103.

(S11) Vogel, A.; Noack, J.; Hüttman, G.; Paltauf, G. Mechanisms of femtosecond laser nanosurgery of cells and tissues. *Appl. Phys. B* **2005**, *81*, 1015–1047.

(S12) Grand, D.; Bernas, A.; Amouyal, E. Photoionization of aqueous indole: Conduction band edge and energy gap in liquid water. *Chem. Phys.* **1979**, *44*, 73–79.

(S13) Nikogosyan, D. N.; Oraevsky, A. A.; Rupasov, V. I. Two-photon ionization and dissociation of liquid water by powerful laser UV radiation. *Chem. Phys.* **1983**, *77*, 131–143.

(S14) Keldysh, L. V. Ionization in the field of a strong electromagnetic wave. *Jetp* **1965**, *20*, 1307–1314.

(S15) Morgan, C. G. Laser-induced breakdown of gases. *Reports Prog. Phys.* **2001**, *38*, 621–665.

(S16) Mainfray, G.; Manus, G. Multiphoton ionization of atoms. *Reports Prog. Phys.* **1991**, *54*, 1333–1372.

(S17) Belenov, E. M. Cascade ionization of a gas by a powerful ultrashort pulse of light. *Sov. Phys. JETP-USSR* **1969**, *29*, 772.

(S18) DeMichelis, C. Laser induced gas breakdown: A bibliographical review. *IEEE J. Quantum Electron.* **1969**, *5*, 188–202.

(S19) Razer, Y. P. Breakdown and Heating of Gases Under the Influence of a Laser Beam. *Sov. Phys. Uspekhi* **1966**, *8*, 650–673.

(S20) Linz, N.; Freidank, S.; Liang, X.; Noack, J.; Paltauf, G.; Vogel, A. Roles of tunneling, multiphoton ionization, and cascade ionization for optical breakdown in aqueous media. *AFOSR Int. Res. Initiat. Proj. SPC 053010 / EOARD* **2009**, 0–206, http://www.dtic.mil/dtic/tr/fulltext/u2/a521817.pdf.

(S21) Liang, X.; Linz, N.; Noack, J.; Vogel, A. Modelling of optical breakdown in dielectrics including thermal effects relevant for nanosecond pulses and sequences of ultra-short laser pulses. Lasers and Electro-Optics 2009 and the European Quantum Electronics Conference. CLEO Europe - EQEC 2009. European Conference on. 2009; pp 1–1, http://dx.doi.org/10.1109/CLEOE-EQEC.2009.5191546.

(S22) Boulais, É.; Lachaine, R.; Meunier, M. Plasma Mediated off-Resonance Plasmonic Enhanced Ultrafast Laser-Induced Nanocavitation. *Nano Lett.* **2012**, *12*, 4763–4769.

(S23) Zolotovitskii, Y. M.; Korshunov, L. I.; Benderskii, V. A. Electron work function from metals in a liquid dielectric. *Bull. Acad. Sci. USSR Div. Chem. Sci.* **1972**, *21*, 760–763.

(S24) Bechtel, J. H.; Lee Smith, W.; Bloembergen, N. Two-photon photoemission from metals induced by picosecond laser pulses. *Phys. Rev. B* **1977**, *15*, 4557–4563.

(S25) Bulgakova, N. M.; Stoian, R.; Rosenfeld, A.; Hertel, I. V.; Campbell, E. E. B. Electronic transport and consequences for material removal in ultrafast pulsed laser ablation of materials. *Phys. Rev. B* **2004**, *69*, 054102.

(S26) Logothetis, E. M.; Hartman, P. L. Laser-Induced Electron Emission from Solids: Many-Photon Photoelectric Effects and Thermionic Emission. *Phys. Rev.* **1969**, *187*, 460–474.

(S27) Bisker, G.; Yelin, D. Noble-metal nanoparticles and short pulses for nanomanipulations: theoretical analysis. *J. Opt. Soc. Am. B* **2012**, *29*, 1383.

(S28) Docchio, F. Lifetimes of Plasmas Induced in Liquids and Ocular Media by Single Nd:YAG Laser Pulses of Different Duration. *Europhys. Lett.* **2007**, *6*, 407–412.

(S29) Johnson, P. B.; Christy, R. W. Optical Constants of the Noble Metals. *Phys. Rev. B* **1972**, *6*, 4370–4379.

(S30) Kreibig, U.; Vollmer, M. *J. Am. Chem. Soc.*; Springer Series in Materials Science 25; Springer Berlin Heidelberg: Berlin, Heidelberg, 1995; Vol. 25.

(S31) Coronado, E. A.; Schatz, G. C. Surface plasmon broadening for arbitrary shape nanoparticles: A geometrical probability approach. *J. Chem. Phys.* **2003**, *119*, 3926.

(S32) Novo, C.; Gomez, D.; Perez-Juste, J.; Zhang, Z.; Petrova, H.; Reismann, M.; Mulvaney, P.; Hartland, G. V. Contributions from radiation damping and surface scattering to the linewidth of the longitudinal plasmon band of gold nanorods: a single particle study. *Phys. Chem. Chem. Phys.* **2006**, *8*, 3540–3546.

(S33) COMSOL AB, RF Module: User's Guide. *COMSOL Multiphysics 5.2* **2016**,

(S34) Beuthan, J.; Minet, O.; Helfmann, J.; Herrig, M.; Müller, G. The spatial variation of the refractive index in biological cells. *Phys. Med. Biol.* **1996**, *41*, 369–382.

(S35) Chen, J. K.; Beraun, J. E. Numerical Study of Ultrashort Laser Pulse Interactions With Metal Films. *Numer. Heat Transf. Part A Appl.* **2001**, *40*, 1–20.

(S36) Kennedy, P.; Boppart, S.; Hammer, D.; Rockwell, B.; Noojin, G.; Roach, W. A first-order model for computation of laser-induced breakdown thresholds in ocular and aqueous media. II. Comparison to experiment. *IEEE J. Quantum Electron.* **1995**, *31*, 2250–2257.

(S37) Plech, A.; Kotaidis, V.; Grésillon, S.; Dahmen, C.; von Plessen, G. Laser-induced heating and melting of gold nanoparticles studied by time-resolved x-ray scattering. *Phys. Rev. B* **2004**, *70*, 195423.
